# Effects of Training Status and Exercise Mode on Global Gene Expression in Skeletal Muscle

**DOI:** 10.3390/ijms222212578

**Published:** 2021-11-22

**Authors:** Daniel A. Bizjak, Martina Zügel, Gunnar Treff, Kay Winkert, Achim Jerg, Jens Hudemann, Frank C. Mooren, Karsten Krüger, Andreas Nieß, Jürgen M. Steinacker

**Affiliations:** 1 Division of Sports and Rehabilitation Medicine, Department of Internal Medicine II, University of Ulm, 89075 Ulm, Germany; martina.zuegel@gmail.com (M.Z.); gunnar.treff@uni-ulm.de (G.T.); kay.winkert@uniklinik-ulm.de (K.W.); achim.jerg@uniklinik-ulm.de (A.J.); Juergen.Steinacker@uniklinik-ulm.de (J.M.S.); 2 Department of Sports Medicine, University Hospital Tübingen, 72074 Tübingen, Germany; jens.hudemann@med.uni-tuebingen.de (J.H.); andreas.niess@med.uni-tuebingen.de (A.N.); 3 Department of Medicine, Faculty of Health, University of Witten/Herdecke, 58455 Witten, Germany; frank.mooren@uni-wh.de; 4 Department of Exercise Physiology and Sports Therapy, University of Gießen, 35394 Gießen, Germany; Karsten.Krueger@sport.uni-giessen.de

**Keywords:** training status, transcriptional regulation, endurance exercise, strength exercise, microarray, pathway analysis, molecular muscle adaptations

## Abstract

The aim of this study was to investigate differences in skeletal muscle gene expression of highly trained endurance and strength athletes in comparison to untrained individuals at rest and in response to either an acute bout of endurance or strength exercise. Endurance (ET, *n* = 8, VO_2_max 67 ± 9 mL/kg/min) and strength athletes (ST, *n* = 8, 5.8 ± 3.0 training years) as well as untrained controls (E-UT and S-UT, each *n* = 8) performed an acute endurance or strength exercise test. One day before testing (Pre), 30 min (30′Post) and 3 h (180′Post) afterwards, a skeletal muscle biopsy was obtained from the m. vastus lateralis. Skeletal muscle mRNA was isolated and analyzed by Affymetrix-microarray technology. Pathway analyses were performed to evaluate the effects of training status (trained vs. untrained) and exercise mode-specific (ET vs. ST) transcriptional responses. Differences in global skeletal muscle gene expression between trained and untrained were smaller compared to differences in exercise mode. Maximum differences between ET and ST were found between Pre and 180′Post. Pathway analyses showed increased expression of exercise-related genes, such as nuclear transcription factors (NR4A family), metabolism and vascularization (PGC1-α and VEGF-A), and muscle growth/structure (myostatin, IRS1/2 and HIF1-α. The most upregulated genes in response to acute endurance or strength exercise were the NR4A genes (NR4A1, NR4A2, NR4A3). The mode of acute exercise had a significant effect on transcriptional regulation Pre vs. 180′Post. In contrast, the effect of training status on human skeletal muscle gene expression profiles was negligible compared to strength or endurance specialization. The highest variability in gene expression, especially for the NR4A-family, was observed in trained individuals at 180′Post. Assessment of these receptors might be suitable to obtain a deeper understanding of skeletal muscle adaptive processes to develop optimized training strategies.

## 1. Introduction

What makes the ‘perfect’ athlete? The chance of an individual possessing a ‘perfect’ genotype for either endurance or strength sports is lower than 1 in 20 million. Moreover, the increasing number of associated polymorphisms decreases the odds accordingly [1]. The genotype dependency of the adaptive response to exercise training and the associated exercise performance is well documented. A twin study with mono- and dizygotic twins, for example, reported an heritability contribution to VO_2_max of 80–90% [2]. This finding was confirmed by the HERITAGE family study, determining the role of genotype for cardiovascular, metabolic, and hormonal responses to aerobic exercise training [3]. Moreover, a considerable variability of individual responses to a standardized endurance or strength training program has been reported [4,5,6]. Lortie et al. showed that the individual differences in the response to a standardized 20-week aerobic training ranged from 5–88% [4], also suggesting that particular genotypes might be more responsive to training than others.

Since the advent of genomic expression analysis, several attempts have been undertaken to link candidate genes and polymorphisms to exercise performance or adaptability to training, estimating the influence of nature (genes) versus nurture (environment) [7,8]. However, exercise performance is a complex trait and there is no direct relationship between genotype and phenotype. Other factors, such as lifestyle, nutrition, and epigenetic changes, considerably affect non-heritable genomic expression [9]. Current studies suggest an influence of phenotypic heritability essential to elite human performance above 50% but below 100% [7], revealing that environmental contributions are an essential part of athletic success.

Transcriptional variability between individuals further complicates the determination of general exercise-response genes for a specific type of exercise. Both epigenetic [10] and genomic [11] adaptions in exercising subjects are highly interindividual, resulting in transcriptional variability and different (muscle) phenotype differentiation. The transcriptome is even affected by acute and long-term exercise intervention [12], as well as the timing of biosampling during and after an acute exercise [13]. Although several similar gene transcriptional adaptations are observed with regular exercise in the exercising muscle [14], a recent meta-analysis showed that the adaptation strategies of skeletal muscle to different levels of physical activity (decreased or increased) may vary in direction and demonstrate qualitative differences that are associated with the activation of different sets of transcription factors [12].

There is a rather complex regulatory network involved in gene expression post-transcription and post-translation events in response to exercise training [15]. Therefore, rare genetic variants are likely to have a smaller impact on physical performance compared to other physiological factors [16]. The complexity of the human response to exercise warrants integrated, global, ‘omics‘ (e.g., genomics, transcriptomics, proteomics, metabolomics, secretomics) approaches to resolve the integrative networks underlying the response to exercise [17,18,19]. Technological advances in genome-wide association, as well as global profiling of gene expression (transcriptomics), have contributed to our understanding of genes, pathways and networks involved in athletic talent and trainability. Thus, microarray technology is increasingly used to determine expression profiles in different populations to investigate health and disease mechanisms and exercise interventions [20,21,22]. The Affymetrix chip array platform in particular has been used in previous studies for profiling of exercise-related gene expression [20,23,24,25].

Athletic talents are normally identified on the field, but when higher and specific training loads are applied, the responses of those talents are various and the understanding of response and resistance to training is limited [8,26,27]. Exercise-induced transcripts [19], molecular markers and proteomics might complement the current conventional approaches to analyze training [28].

In this study, we examined highly endurance- and strength-trained athletes and compared them to untrained individuals to investigate skeletal muscle gene expression (1) at rest prior to exercise, (Pre) representing the basal expression level; and (2) 30 min (30′ Post) and 180 min (180′ Post) after an acute bout of either endurance or strength exercise to determine exercise mode-specific transcriptional regulation processes.

## 2. Results

### 2.1. Participant Characteristics

A detailed participant and group categorization is provided in the Materials and Methods section and in Table 1. In short, participants were categorized into endurance trained (ET), strength trained (ST) and two untrained groups (UT) (total number *n* = 32, 8 participants in each group). Trained individuals in ET and ST had been training for at least three years. UT either performed an acute bout of endurance (E-UT) or strength exercise (S-UT) and subjects of all groups met the following criteria: male, 18–30 years of age, body mass index (BMI) 18.5–28.5 kg/m², non-smoker, no chronic diseases.

ET showed a mean VO_2_max of 67± 9 mL/kg/min and ST had mean strength training experience of 5.8 ± 3.0 training years.

### 2.2. Effects of Training Status on Global Skeletal Muscle Gene Expression Levels at Rest

In total, 49,386 genes (defined as coding sequences including different isoforms) were analyzed with the Affymetrix^®^ HG-U219 gene array. At baseline, ET vs. E-UT showed 409 differently expressed genes (158 up-regulated, 251 down-regulated). ST vs. S-UT exhibited 366 differently expressed genes (303 up-regulated, 63 down-regulated). 1526 genes (558 up-regulated, 968 down-regulated) were differentially expressed at rest between ET vs. ST. Training status (trained vs. untrained) therefore showed an influence on transcriptional regulation at rest, however, exercise mode (ET vs. ST) in trained individuals exhibited the stronger effect on basal skeletal muscle gene expression (Table 2). A top 20 of differentially expressed genes between the respective groups according to highest fold change is provided in Supplementary Materials.

**Figure 1:**
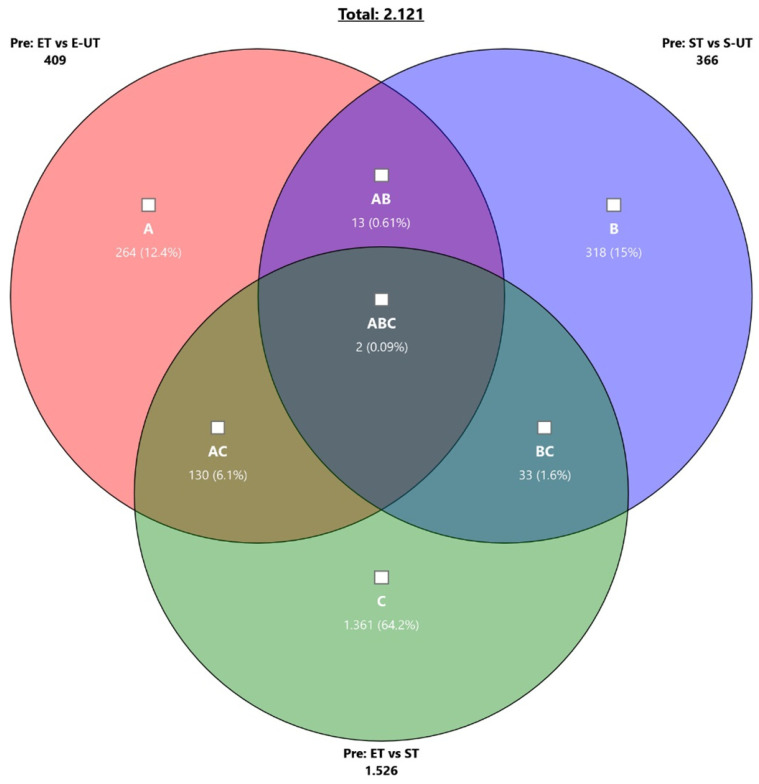
Venn-diagram of the differential expressed genes regarding the comparison of ET vs. E-UT (circle **A**), ST vs. S-UT (circle **B**) and ET vs. ST (circle **C**) at baseline time point Pre. The overlap of ET/E-UT and ST/S-UT in differently expressed genes was only 0.61% (13 genes, **AB**), whereas the highest overlap was seen between ET/E-UT and ST with 6.1% (130 genes, **AC**).

An overview of the differences and the respective gene expression overlap at rest is provided in Figure 1 and Table 3.

### 2.3. Effects of Training Status on Global Skeletal Muscle Gene Expression Levels at Pre, 30′ Post and 180′ Post

**Figure 2:**
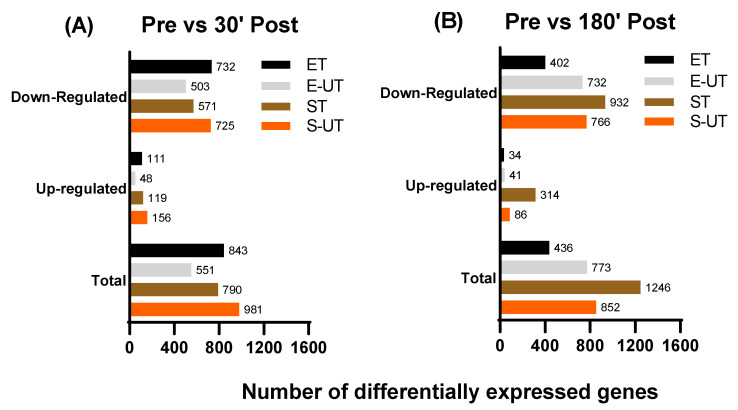
Number of differentially expressed genes in response to the exercise test (endurance or strength) (**A**) Pre vs. 30′ Post and (**B**) Pre vs. 180′ Post. The total number of differentially expressed genes in skeletal muscle of highly endurance-trained (ET) participants is higher at Pre vs. 30′ Post compared to Pre vs. 180′ Post, while a higher number of differentially regulated genes is noted at Pre vs. 180′ Post for ST. There is no major difference between Pre vs. 30′ Post or Pre vs. 180′ Post for the untrained control groups (E-UT and S-UT). 30′ Post vs. 180′ Post results are provided in Supplementary Materials.

Comparison between ET/ST and the control groups E-UT/S-UT revealed overall highest differences between Pre and 30′ Post as well as Pre vs. 180′ Post (Figure 2), while the difference between 30′ Post and 180′ Post was comparatively smaller: only 172 differently expressed genes were detected for ET (107 up, 65 down), 151 for E-UT (24 up, 127 down), 191 for ST (92 up, 99 down) and 121 for S-UT (27 up, 94 down), respectively. Therefore, a special focus was laid on the comparison between Pre and 180′ Post. The highest overlap (41.4%) as well as highest total number of differentially expressed genes (1484 genes) was observed ST vs. S-UT. The lowest total number was seen ET vs. E-UT (877 genes), while the overlap percentage of ET and ST was only 20.9% (Venn diagrams with gene overlaps are provided in S2. 30′ Post results are additionally provided in S2.

The total number of differentially expressed genes in skeletal muscle of ET was higher at Pre vs. 30′ Post (843 genes) compared to Pre vs. 180′ Post (436 genes), while for ST a higher number of differentially regulated genes is noted at Pre vs. 180′ Post (1246 genes) compared to Pre vs. 30′ Post (790 genes).

For the untrained groups, a higher number of differentially regulated genes was found for E-UT at Pre vs. 180′ Post (773 genes) compared to Pre vs. 30′ Post (551 genes) while an approximately equal number of differentially expressed genes was found for S-UT at Pre vs. 30′ Post (981 genes) and Pre vs. 180′ Post (852 genes).

### 2.4. Effects of Acute, Mode-Specific Exercise on Expression Levels of Selected Genes Involved in Skeletal Muscle Transcription, Metabolism, Muscle Growth/Structure and Vascularization

#### Pathway Analysis

Pathway analysis (Transcriptome Analysis Console 4.0 Software Thermo Fisher Scientific, Waltham, MA, USA, WikiPathways) of differently expressed genes involved in energy metabolism identified the following pathways: VEGFA-/VEGFR2 signaling, Adipogenesis-, Nuclear receptors meta- and PI3K-Akt signaling pathway provided by Wiki-Pathways (Supplementary Materials). Further analyses led to the identification of the following exercise-related genes: NR4A family (NR4A1, NR4A2, NR4A3), VEGF-A, HIF-1α, PGC-1α, IRS1, IRS2 and Myostatin. The respective pathways were identified by differently expressed gene counts and fold change significance. The NR4A orphan receptor family in particular showed an increased expression after the exercise test in all groups, which was most pronounced 180 min after exercise in all groups (30–68-fold increase vs. Pre). The pronounced effect for the untrained individuals (E-UT and S-UT) was also observed for most of the other analyzed genes. An overview of the regulatory effects for the respective training groups is provided in Table 3.

### 2.5. Exercise Related Gene Expression Regulation in NR4A Family, Metabolism and Vascularization, and Muscle Growth/Structure after Acute Exercise

**Figure 3:**
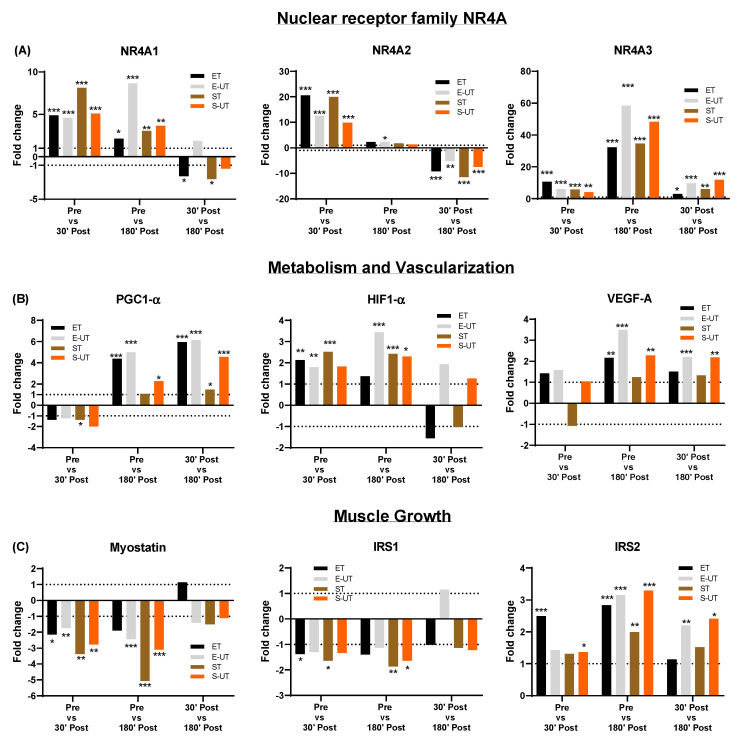
Fold change of genes involved in (**A**) nuclear transcription (NR4A family), (**B**) metabolism and vascularization (PGC1-α, HIF-1α and VEGF-A) and (**C**) muscle growth/structure (myostatin, IRS1 and IRS2) at Pre (baseline) vs. 30′ Post, Pre vs. 180′ post and 30′ Post vs. 180′ Post. The respective genes were selected after pathway analysis of exercise related metabolic pathways. Dotted lines signify the fold-change of 1 and −1. Significance level was set at * *p* ≤ 0.05, ** *p*≤ 0.01, *** *p* ≤ 0.001.

A detailed graphical presentation of the up- and down-regulation in the respective groups at the different time points is shown in Figure 3. While there is mostly an upregulation in the NR4A family over time and groups, muscle growth regulator gene Myostatin was downregulated as well as IRS1. IRS2 was upregulated at all time points, whereas the comparison of the time points regarding genes involved in metabolism and vascularization revealed a training status/group specificity. The different biopsy time points showed a time dependency of changes in expression in the respective genes, rather than an exercise group specific change. Whereas NR4A1 and NR4A2 showed highest expression changes 30′ Post, the greatest expression changes for NR4A3, PGC-1α, VEGF-A and IRS2 were observed 180′ Post compared to Pre. HIF-1α, myostatin and IRS1 expression exhibited similar changes either 30′ Post or 180′ Post.

## 3. Discussion

The groups of endurance and strength athletes and their untrained control groups responded differently to acute exercise on the genetic level. While differences in the number of regulated genes were not—or only moderately—associated with training status prior to exercise, the mode of exercise (i.e., strength or endurance) significantly affected overall differences in skeletal muscle gene expression within the trained groups. This study aimed to allow the analysis of relevant performance-related transcripts to create a library of genes relevant for acute exercise and training responses, so great effort was spent on the physiological characterization of our subjects, with the inclusion of only highly trained subjects, who differed sharply in their performance and training history compared to untrained individuals.

Pathway analyses showed increased expression of exercise-related genes, such as nuclear transcription factors (NR4A family), metabolism and vascularization (PGC-1α and VEGF-A), and muscle growth/structure (myostatin, IRS1/2 and HIF1-α). The most significantly upregulated genes in response to acute endurance or strength exercise were the NR4A genes (NR4A1, NR4A2, NR4A3). NR4A1 increased 30′ Post as well as 180′ Post exercise between two to eightfold in both ET and ST as well as untrained controls. Even higher expression increases were observed for NR4A2 and NR4A3. NR4A2 increased 30′ Post between 10–20-fold, and NR4A3 even increased between 30–68-fold 180′ after acute exercise. The highest expression increase was observed in skeletal muscle samples of E-UT at 180′ Post (a 68.33-fold increase).

### 3.1. Exercise Mode Rather Than Training Status Is Associated with Differences in Global Skeletal Muscle Gene Expression at Rest between Trained and Untrained Participants

Surprisingly, there were comparatively smaller differences in skeletal muscle gene expression profiles between trained and untrained individuals at rest (ET vs. E-UT 409 differently expressed genes, ST vs. S-UT 366 differently expressed genes) with regard to basal differences between ET and ST (1526 differently expressed genes). The analysis of the 20 genes with highest fold change differences between the groups revealed muscle specific differences in differentially expressed genes like myostatin (ET vs. E-UT), actin in cardiac muscle (ST vs. S-UT), or myosin heavy chain 1 in skeletal muscle (ET vs. UT) (S1). Chapman et al. conducted a global skeletal muscle transcriptomic analysis of long-term endurance- and strength-trained males and females (>15 years training history). Results showed that, compared with age-matched controls, decades-long endurance training in particular shifts the resting muscle transcriptome significantly, resulting in diminished sex differences and altered gene expression patterns associated with attenuation of metabolic disease [29]. In particular, genes involved in cellular respiration and tricarboxylic acid metabolism were significantly enhanced in long-term endurance-trained men and women, while strength training did not have a large effect on the resting skeletal muscle transcriptome. Interestingly, regular endurance training over 6–12 months shifted gene expression patterns in individuals with impaired metabolism towards the transcriptome found in long-term endurance-trained individuals, including genes involved in oxidative metabolism, mitochondrial structure, protein metabolic processes and vascular development [29]. One of the reasons for the conflicting results may be that participants in our study were younger (18–30 years) compared to participants in the study by Chapman et al. (34–53 years, at least 15 years of training), and therefore had a shorter training history. Thus, interestingly, the exercise mode rather than the training status seems to influence basal gene expression adaptations, which underlines training responsive up- or downregulation of specific exercise-related pathways [30].

### 3.2. Response of Exercise-Related Genes to Acute Exercise in Trained and Untrained Individuals

As mentioned above, the most significantly upregulated genes in response to acute endurance or strength exercise were the NR4A genes. The NR4A family belongs to the orphan receptor family and includes three members, namely Nur77 (NR4A1), Nurr1 (NR4A2) and Nor1 (NR4A3) [31]. They regulate the expression of genes which participate in several biological functions, including metabolism (particularly glucose and fatty acid utilization genes in skeletal muscle), immunity, cellular stress, memory and insulin sensitivity [31,32]. As early-response genes without endogenous ligands, their expression is induced by diverse stimuli, e.g., exercise-related activators of cAMP and protein kinase signaling, mechanical stress or physiological activity [33,34]. Catoire et al. observed up-regulated NR4A expression after acute one-legged exercise in middle aged individuals compared to the respective non-exercise leg [35], while Rundqvist et al. showed increased NR4A3 expression after acute sprint exercise [36]. The results of Kawasaki suggest that local contractile activity is required for increased NR4A expression during exercise and that contractile activity-induced increases may be mediated by AMPK activation [37]. Hence, the NR4A-pathway may be a crucial regulatory mechanism for enhanced metabolic performance regulation for either endurance or strength exercise and a prospective target for expression monitoring on the genomic and protein level as marker for athletic performance.

While NR4As might be especially involved in energy metabolism, a further important factor for performance capacity is molecular respiratory performance of tissues, erythrocytes, and mitochondria. Keller et al. tried a first approach to classify proangiogenic and tissue developmental networks after endurance exercise with respect to high and low responders [38]. They observed a significant up-regulation of genes involved in cardiovascular development in both groups, but to a greater extent in high responders to aerobic exercise training. Additional rat experiments of this group revealed possible beneficial gene adaptations in oxygen sensing and angiogenesis. One key player of angiogenesis is VEGF, the expression of which can be induced by different exercise-related stimuli like hypoxia or increased vasodilatory activity [39]. HIF-1α acts as one VEGF-regulator under hypoxia [39]. Both VEGF and HIF-1α were upregulated after acute exercise in our trained and untrained participants, where E-UT showed the most prominent and simultaneous fold changes in VEGF and HIF-1α 180′ Post. This might be related to the training status as well as the exercise stimuli, as the hypoxia-induced effect of angiogenesis can be expected to be attenuated, either due to the continuous hypoxia training in ET, or the less hypoxic exercise in strength training.

The effects of chronic endurance and strength training on genomic adaptations was examined by Stepto et al. in well trained endurance and strength athletes at rest [40]. A comparison of the global m. vastus lateralis genome showed distinctive differences in mitochondrial capacity, fat and carbohydrate gene clusters, which were upregulated in endurance athletes and even correlated with peak oxygen consumption. Likewise, our acute exercise led to increased expression of genes involved in mitochondrial and erythrocytic biogenesis, i.e., PGC-1α and, as mentioned above, HIF-1α in endurance athletes. Again, the fold change was even more pronounced in untrained controls and, interestingly, the same was observed for the NR4A family. All these observations could of course be due to the unfamiliar exercise in the respective untrained control groups, where the acute exercise may have induced an unaccustomed magnitude of stress and intensity, whereas our athletes may exhibit very low sensitivity to this familiar exercise, as shown previously [22].

Another identified pathway involved in the response to exercise was adipogenesis and muscle growth. It is assumed that IRS1/IRS2 dysregulation plays a crucial role in the development of obesity and diabetes [41,42]. Both IRS1 and IRS2 were shown to be regulated by PGC-1α, the “master-regulator” of numerous pathways involving both metabolism and overall energy homeostasis (e.g., mitochondrial biogenesis) [43]. Upregulation of PGC-1α expression led to IRS1 down-regulation and IRS2 up-regulation in hepatic mouse cells [42]. Besse-Patin and co-workers suggested that the IRS1:IRS2 ratio supports the specification or amplification of specific pathways downstream of insulin signaling within different metabolic contexts, like shutdown of gluconeogenesis over enhancing lipogenesis (high IRS2), or favoring the lipogenic arm of insulin signaling (high IRS1) for overall energy control i.a. in adipogenesis. Our microarray data confirm these observations of reciprocal control, as the upregulation of PGC-1α 30′ Post and 180′ Post showed concomitant decreased IRS1 and increased IRS2 expression. At this point, an expected increased fold change of PGC-1α was observed in the trained and untrained endurance groups due to the aerobic exercise mode, whereas the untrained strength participants in particular showed an increased response, again leading to the assumption of unaccustomed training load.

The adipogenesis pathways, in addition to their key role in insulin signaling, are also important mechanisms for the regulation of muscle mass, as it was shown that IRS1 and IRS2 dysregulation can contribute to skeletal muscle loss and wasting [44]. Experiments in rodents by Long et al. examining the control of Akt-mTor initiated muscle growth supports this observation, as IRS knock-out mice exhibited significantly reduced skeletal muscle growth and Akt-mTOR signaling [45]. In this context, an important negative regulator of skeletal muscle mass deposition is myostatin. It is a member of the TGF-β superfamily of growth and differentiation factors that act as muscle growth regulators [46,47]. Mutations in the myostatin gene can result in increased bulky muscle mass with advantages in elite strength competitions [48,49]. Myostatin expression was significantly downregulated in our participants both 30′ Post and 180′ Post. ST showed the highest fold downregulation change of all groups supporting the anti-catabolic effect and regulation of strength training on the mRNA transcription level. As the downregulation was further increased 180′ Post, a later sampling time point would have given even more valuable insights into how long the acute strength exercise effect might prevail.

A retrospective gene array analysis by Bonafiglia et al. showed that acute resistance exercise led to a response of 1290 genes that were mainly involved in pathways regulating muscle adaptations [25]. On the other hand, a study by Damas et al. [50] showed that training status (10-week resistance exercise training) was associated with different muscle transcriptome responses. The responses to acute resistance exercise in untrained muscles before the training intervention involved upregulation of stress, damage, remodeling, inflammation and protein turnover, whereas in trained muscles after 10-weeks of resistance exercise, changes in energy metabolism, antioxidant-/immune-regulation, muscle contraction, development and growth was noted [50]. These studies confirm our results showing that individual transcriptional responses are dependent on performance status and exercise mode.

### 3.3. Limitations and Future Aims

Our results and those of other groups provide increasing evidence that the timing of muscular biopsy sampling for microarray analysis is crucial for the monitoring of genetic expression profiles. Gene response times to exercise can be very fast and muscle biopsies are often restrained to certain time points due to invasive and technical restrictions. Nevertheless, our data contribute to the identification of early response genes related to acute endurance and strength exercise.

As Vissing and Schjerling highlighted, the combined gene adaptation examination of strength, endurance and untrained individuals during an intervention study seems necessary, as some mechanisms are responding essentially differently to different exercise modes, whereas others may exhibit overlap in responses [22].There is a certain (genomic) individual variability to respond to endurance or strength training, but the general view of “responders and non-responders” has been challenged lately [51].

Although microarray application technologies have greatly improved during the last decade and are now available for a broader field of scientists, routine applications in larger study populations are still limited because of cost, in addition to practical limitations and results interpretation. The cooperation between bioinformaticians and sports medicine scientists is crucial to distinguish between biologically relevant transcription signal interpretation, e.g., in the case of different isoform detection of the examined gene. Nevertheless, the identification of novel and prospective genetic targets to monitor training load and for talent identification may support the inclusion of exercise-specific pathway analyses, which is still lacking in genome mapping. The optimal timing of biosampling/muscle biopsies in particular has to be elucidated for respective gene analysis, as each gene differs in its expression profile in response to different stimuli [13], and sex-specific adaptations also need to be considered [52].

We share the opinion of Bouchard et al. that the combination of genomics, epigenomics and transcriptomics in combination with large observational but preferably experimental study designs is needed to increase replication and validation of the interdependence of transcriptional regulation, transcriptional response, and exercise, which has to be met with large-scale collaborative and multicenter research programs [15]. Until then, genomic talent identification to support trainers and athletes alike will be difficult to achieve.

## 4. Materials and Methods

### 4.1. Participants

This study aimed to compare physiological and skeletal muscle molecular responses between highly endurance- and strength-trained and untrained participants at rest and in response to an acute bout of either endurance or strength exercise. In total, 72 participants were recruited at the three study centers, the Departments of Sports Medicine at the Universities of Ulm, Tübingen and Gießen (Germany). As stated above, 32 participants (*n* = eight in each group) were further categorized into endurance trained ET, strength trained ST, and two untrained groups UT, which either performed an acute bout of endurance (E-UT) or strength exercise (S-UT).

Subjects were categorized into the four groups based on a sports-specific exercise anamnesis, aerobic capacity (VO_2_max) and their one repetition maximum (1-RM) serving as a surrogate of muscular strength (see Table 4).

ET had a VO_2_max > 57 mL/min/kg and had to fulfill two out of three strength requirements (<3.5-fold body mass in leg press, <1.0-fold body mass in bench press and <1.0-fold body mass in bench pull). Strength athletes were required to have a VO_2_max < 47 mL/min/kg and needed to fulfill the criterion for leg press as well as for either bench press OR bench pull OR both (1 + 1 of 2; >3.5-fold body weight in leg press, >1.0-fold body weight in bench press and/or bench pull). Untrained subjects had to have a VO_2_max < 47 mL/min/kg and needed to fulfill at least two out of three criteria for inclusion (two out of three; <2.5-fold body weight in leg press, <1.0-fold body weight in bench press, <1.0-fold body weight in bench pull).

### 4.2. Study Design

The study consisted of two separate examination days. On the first day, after the physical examination by a licensed physician, a cardiopulmonary exercise test (CPX) was performed on a bicycle ergometer (Lode Excalibur, Lode, The Netherlands). The CPX consisted of an incremental step test (increment of 25 watts each 3 min) with additional lactate diagnostics to determine VO_2_max and lactate thresholds. Afterwards, a maximum strength test was performed. The maximum strength test consisted of three exercises in order to distinguish between trained strength athletes and untrained controls based on the one-repetition maximum (1-RM) (Table 4).

The second day was preceded by a 72-h period where participants abstained from their usual training or any physical exercise. On the morning of the acute exercise test (either strength or endurance), each subject received a standardized breakfast. Drinking water was allowed ad libitum before, during, and after exercise. One hour after the end of the acute exercise test, each subject received a cheese roll.

**Figure 4:**
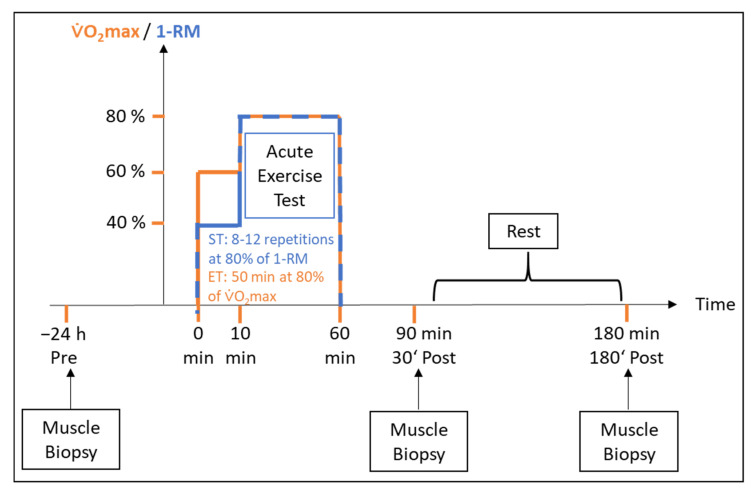
Schematic illustrating the acute exercise testing procedures. Prior to the exercise test, a skeletal muscle biopsy was taken at rest. For the acute endurance exercise test, participants cycled for 50 min at 80% of VO_2_max. The strength test consisted of 8–12 repetitions at 80% of 1-RM (bench pull, knee extension, bench press, leg press). Skeletal muscle biopsies were taken prior to exercise (Pre), 30 min (30′ Post) and 180′ post-exercise (180′ Post).

The acute exercise test was performed either in the weight room for ST and S-UT or in a cardiopulmonary exercise lab on a bicycle ergometer for ET and E-UT. Acute strength exercise was performed with a warm-up series at 40% of 1-RM, followed by five series of 8–12 repetitions at 80% of 1-RM on all three training machines (bench pull, bench press, leg press). If necessary, weight reduction was also possible in case eight repetitions were not achieved. Time under tension (TUT) was recorded. This was divided into a 10-min warm-up phase at a mechanical power output corresponding to 60% of VO_2_max, followed by 80% of VO_2_max over 50 min (Figure 4).

### 4.3. Skeletal Muscle Biopsy and Blood Sampling

Skeletal muscle biopsies were performed before (Pre), 30 min after (30′ Post) and 3 h after (180′ Post) the acute exercise test. Skeletal muscle tissue was used for microarray analysis. The muscle biopsy taken at rest in the morning was performed in the middle of the belly of the right musculus vastus lateralis. After thorough disinfection of the injection site, the skin in the corresponding area was anesthetized with local anesthetics (1% mepivacaine). Fine-needle muscle biopsies (Plus Speed; Peter Pflugbeil GmbH, Zorneding, Germany) were subsequently obtained. The obtained tissue pieces were frozen in liquid nitrogen immediately and stored at −80 °C until further processing. The sampling site was manually compressed for 5–10 min after completion of specimen collection and then dressed with a sterile pressure dressing.

For RNA isolation, muscle tissue was incubated for 24 h with RNAlater (QIAGEN GmbH, Hilden, Germany) at 4 °C and then stored in cryotubes at −80 °C until further analysis.

### 4.4. RNA Isolation and Microarray Analysis

Muscle samples were homogenized using a Tissue Ruptor (Qiagen, Venlo, The Netherlands). To obtain RNA, samples were subsequently purified using RNeasy Mini Kit columns (RNeasy Fibrous Tissue Mini kit, Qiagen, Venlo, The Netherlands). The RNA concentration of the samples was measured with the NanoDrop 2000/2000 c full spectrum UV-Vis spectrophotometer (Thermo Scientific™, Rockford, IL, USA) and RNA integrity (RIN) was assessed using the Agilent 2100 Bioanalyzer. The microarray platform used was Affymetrix^®^ HG-U219 gene array chip at MFT Services (Medical Genetics, Tübingen, Germany).

### 4.5. Microarray Data Analysis

The differential gene expression analysis was performed using the Transcriptome Analysis Console (TAC) 4.0 Software (Thermo Fisher Scientific, Waltham, MA, USA). All procedures were carried out according to the manufacturers’ instructions. Robust Multi-array Average (RMA) was used for normalization. Following normalization, differential expression was carried out using eBayes function and One-Way Repeated Measures ANOVA statistical analysis. Gene level fold changes were analyzed at <−1.5 and >1.5 for basal values at PRE. For the examination of the impact of the acute exercise test (30′ Post and 180′ Post), the gene fold threshold was increased to <−2 and >2, since exercise is a potent stimulus to alter gene expression. Statistical significance was set at *p* < 0.05 for all analyses.

### 4.6. Pathway Analysis

We used the TAC pathway analysis tool, which is directly connected to all pathways provided by WikiPathways. Genes above the threshold were sorted by count and significance and upregulation/downregulation visualized in the pathways. The provided pathways were manually screened for pathways involved in response to exercise. Specific target genes with high fold changes were further examined and compared between the different groups.

## 5. Conclusions

To our knowledge, this is the first study to distinguish global genomic responses in skeletal muscle of highly endurance- and strength-trained individuals and their respective control groups at rest and in response to either acute strength or endurance exercise. The greatly increased expression of the NR4A family after acute exercise, which is even more pronounced in untrained individuals, particularly requires further examination to elucidate the role of these orphan receptors as possible targets for training adaptation processes as well as prospective metabolic markers in health and disease. For example, ß-adrenergic stimulation was shown to increase NR4A3 expression [53], which might not only have consequences for performance in athletes suffering from respiratory disorders, but also for elite competitors, where ß-adrenergic stimulation might stimulate NR4A expression, leading to improved energy metabolism and performance compared to healthy individuals. Thus, assessment of these receptors on the genetic and protein level might be suitable to obtain a deeper understanding of skeletal muscle adaptive processes in order to develop optimized training strategies.

## Figures and Tables

**Table 1:** Anthropometric data and maximal oxygen consumption (VO_2_max) of highly endurance-trained (ET), highly strength-trained (ST) and untrained control groups, who completed either an acute endurance (E-UT) or strength session (S-UT). Data are arithmetic mean ± standard deviation. *n* = 8 in each group.

Group	Age [Years]	Body Mass [kg]	Height [cm]	BMI [kg/m^2^]	VO_2_max [L/min]	VO_2_max [mL/kg/min]
ET	25.4 ± 3.8	72.55 ± 5.98	181.1 ± 4.9	22.00 ± 1.11	4.9 ± 0.9	67.2 ± 8.9
E-UT	22.6 ± 2.8	70.48 ± 5.49	180.6 ± 8.1	21.59 ± 1.96	10.2 ± 14.1	32.9 ± 19.1
ST	24.6 ± 4.3	83.63 ± 10.51	177.0 ± 5.9	26.66 ± 3.66	3.6 ± 0.6	43.6 ± 8.1
S-UT	24.3 ± 3.0	75.95 ± 8.30	180.3 ± 6.5	23.06 ± 2.77	3.0 ± 0.3	41.5 ± 5.0

**Table 2:** Overview of the effect of the acute exercise test on the exercise-related genes subdivided into the NR4A family, metabolism and vascularization, and muscle growth/structure.

Metabolic Variables		Training Group Effect	Regulation
Nuclear Transcription Factors (NR4A Family)			
	NR4A1	All	upregulated
	NR4A2	All	upregulated
	NR4A3	All	upregulated
Metabolism and vascularization			
	PGC-1α	ET/E-UT	upregulated
	VEGF	ET/UT	upregulated
Muscle growth/structure			
	Myostatin	All	downregulated
	IRS1	30′ Post: ET 180′ Post: ST/S-UT	downregulated
	IRS2	All	upregulated
	HIF-1α	All	upregulated

**Table 3:** Number of genes differentially expressed between ET vs. E-UT (409 genes), ST vs. S-UT (366 genes) and ET vs. ST (1526).

Comparison	Group 1	Group 2	Time Point	^#^ Per Group	Total	Up	Down
ET vs. E-UT	ET	E-UT	Pre	8	409	158	251
ST vs. S-UT	ST	S-UT	Pre	8	366	303	63
ET vs. ST	ET	ST	Pre	8	1526	558	968

^#^-number of participants per group.

**Table 4:** Inclusion criteria for highly endurance trained (ET) and untrained (E-UT) and highly strength trained (ST) and untrained (S-UT) participants including training volume (h/week), aerobic capacity (VO_2_max), and strength (one repetition max, 1-RM), body weight (BW).

	Endurance Trained (ET)	Endurance Untrained (E-UT)	Strength Trained (ST)	Strength Untrained (S-UT)
Exercise anamnesis:				
Endurance training Strength training	>5 h/Week <1 h/Week	<2 h leisure activities/Week	<1 h/Week >2 × 1.5 h/Week	<2 h leisure activities/Week
VO_2_max:	>57 (mL/min/kg)	<47 (mL/min/kg)	<47 (mL/min/kg)	<47 (mL/min/kg)
One repetition maximum (1-RM):				
Bench pull	<1 × BW	<1 × BW	>1 × BW	<1 × BW
Leg press	<3.5 × BW	<2.5 × BW	>3.5 × BW	<2.5 × BW
Bench press	<1 × KG	<1.5 × KG	>1 × KG	<1.5 × KG
Included participants for microarray analysis	8	8	8	8

The protocols used in this study were approved by the ethics committee (no. 267/11) of Ulm University and align with the Declaration of Helsinki. All participants gave written informed consent to participate in this study.

## Data Availability

All data which are not provided in the main manuscript or Supplementary files can be provided by the corresponding author on reasonable request. Not all data are not publicly available due to privacy restrictions.

## Supplementary Materials

The following data sets are available online at https://www.mdpi.com/article/10.3390/ijms222212578/s1.
