# Supplementary material for: Effects of Training Status and Exercise Mode on Global Gene Expression in Skeletal Muscle
Source: Int J Mol Sci. 2021 Nov 22;22(22):12578. doi: 10.3390/ijms222212578 (PMC8674764; doi:10.3390/ijms222212578)
Supplement: Supplementary file 1 [file ijms-22-12578-s001.zip › Supplemental 2_IJMS.pdf]

Supplemental 1

Table S1. Number of genes differentially expressed between 30` Post and 3h Post in the respective training groups.

Comparison	Group	# per group	Up	Down	Total
30’ Post vs 180’ Post	ET	8	107	65	172
30’ Post vs 180’ Post	E-UT	8	24	127	151
30’ Post vs 180’ Post	ST	8	92	99	191
30’ Post vs 180’ Post	S-UT	8	27	94	121

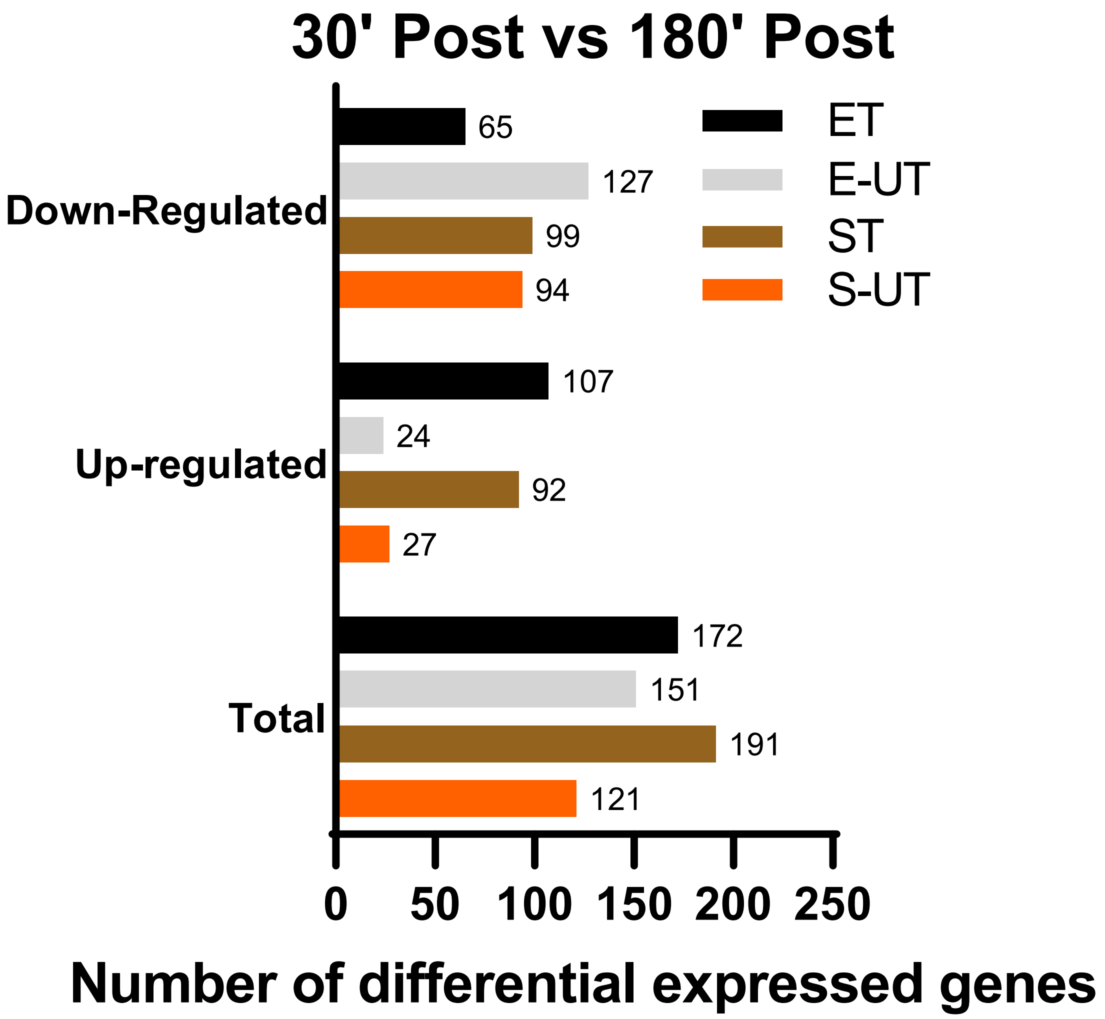

Figure S1: Number of differentially expressed genes in response to the exercise test (endurance or strength) 30’ Post vs 180’ Post. The total number of differentially expressed genes in skeletal muscle of endurance or strength trained (ET and ST) and untrained (E-UT and S-UT) participants is lower compared to Pre vs 30’ Post or to Pre vs. 180’ Post. While ST, S-UT and E-UT showed more down-regulation, ET was the only group where more up- than downregulation was observed.

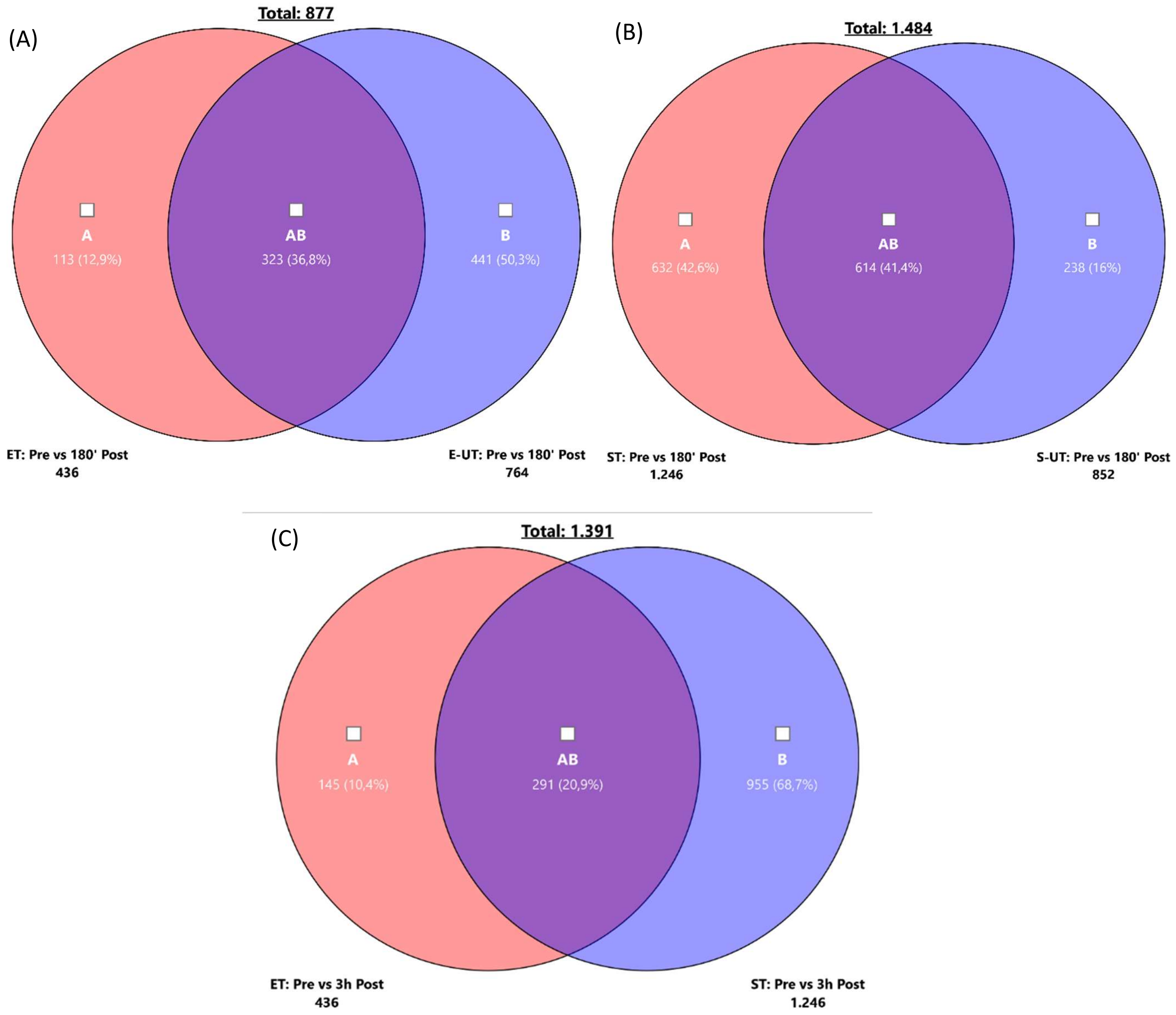

Figure S2: Venn diagrams of (A) ET vs E-UT, (B) ST vs S-UT and (C) ET vs ST regarding differentially expressed genes Pre vs 180' Post. The highest overlap (41.4%) as well as highest total number of differentially expressed genes (1,484 genes) was observed ST vs S-UT (B). The lowest total number was seen ET vs E-UT (877 genes, (A)), while the overlap percentage of ET and ST was only 20.9% (C).
