# Supplementary material for: Effects of Training Status and Exercise Mode on Global Gene Expression in Skeletal Muscle
Source: Int J Mol Sci. 2021 Nov 22;22(22):12578. doi: 10.3390/ijms222212578 (PMC8674764; doi:10.3390/ijms222212578)
Supplement: Supplementary file 1 [file ijms-22-12578-s001.zip › Supplemental 3_IJMS.pdf]

Supplemental 2: Pathway analysis and subsequent sub-analysis of exercise related genes.

The analysis was performed with the Transcriptome Analysis Console (TAC) 4.0 Software (Thermo Fisher Scientific, Waltham, MA USA) and the pathways identified with respect to a) count and b) significance of differentially expressed genes. The presented figures below are provided by Wiki-pathways directly linked to the TAC programme.

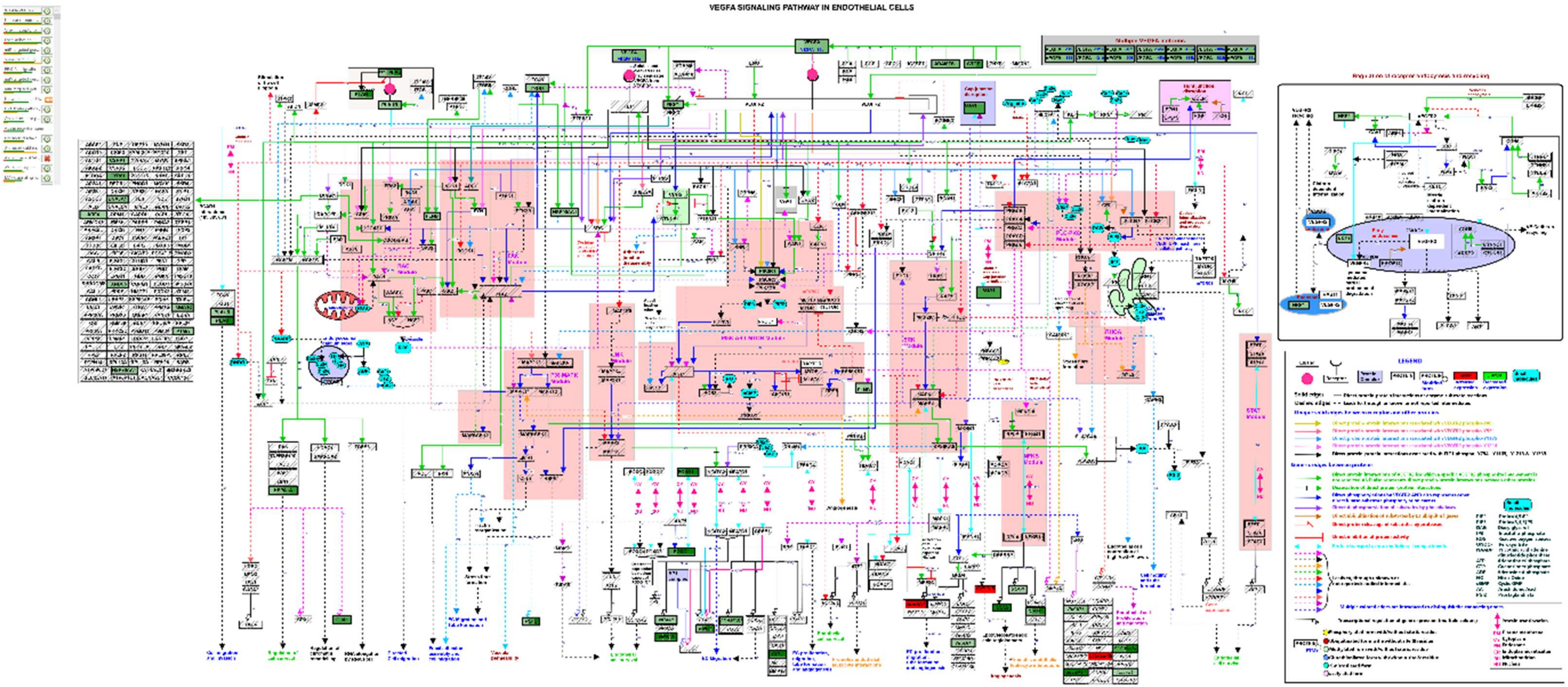

Figure S3: VEGFA-VEGFR2 signaling pathway (Homo sapiens) provided by Wiki-pathways (https://www.wikipathways.org/index.php/Pathway:WP3888). Green marked genes signify a fold change < -4 and red marked genes a fold-change > 4.

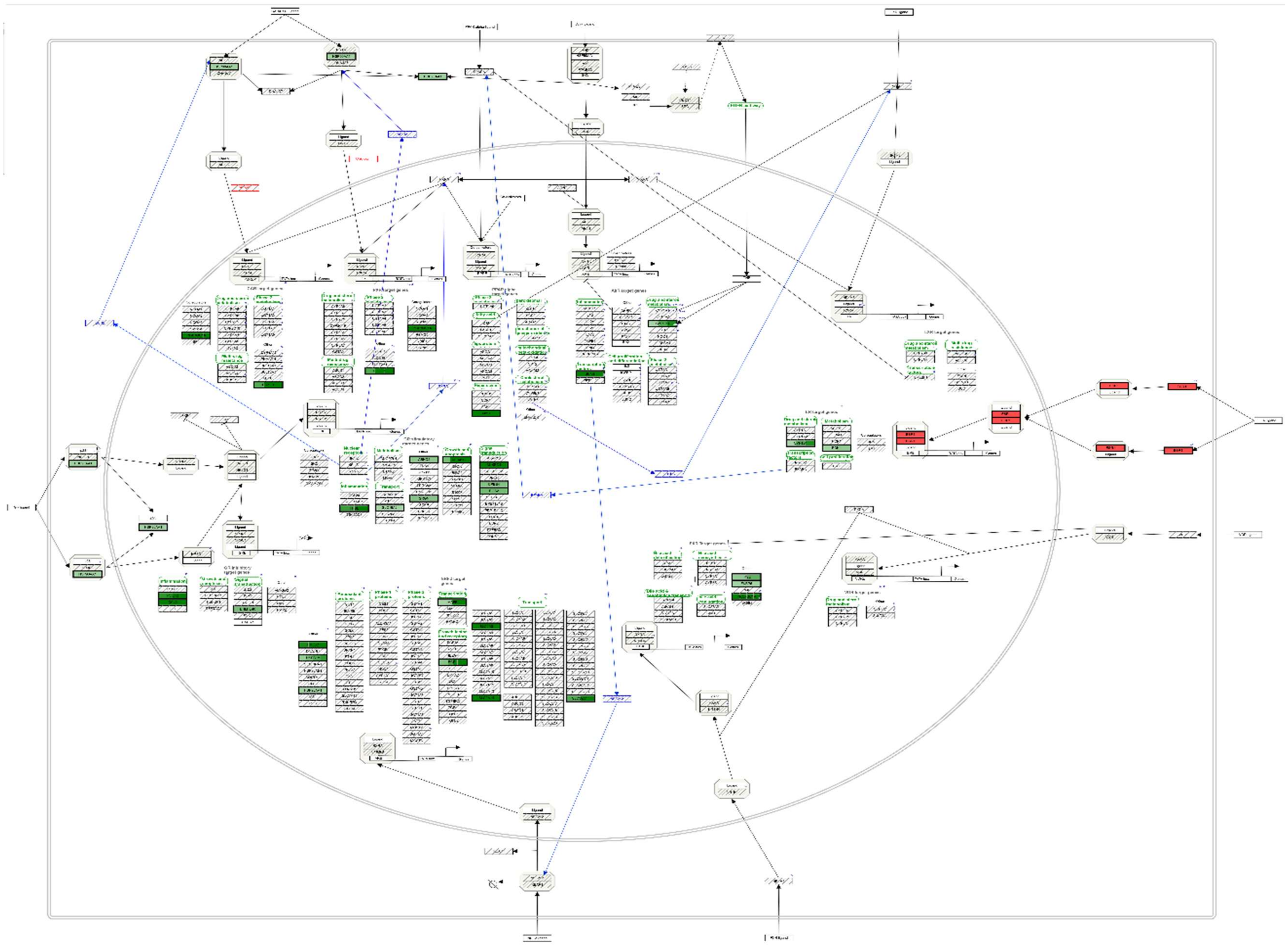

Figure S4: Nuclear receptors meta-pathway (Homo sapiens) provided by Wiki-Pathways (https://www.wikipathways.org/index.php/Pathway:WP2882). Green marked genes signify a fold change < -4 and red marked genes a fold-change > 4.

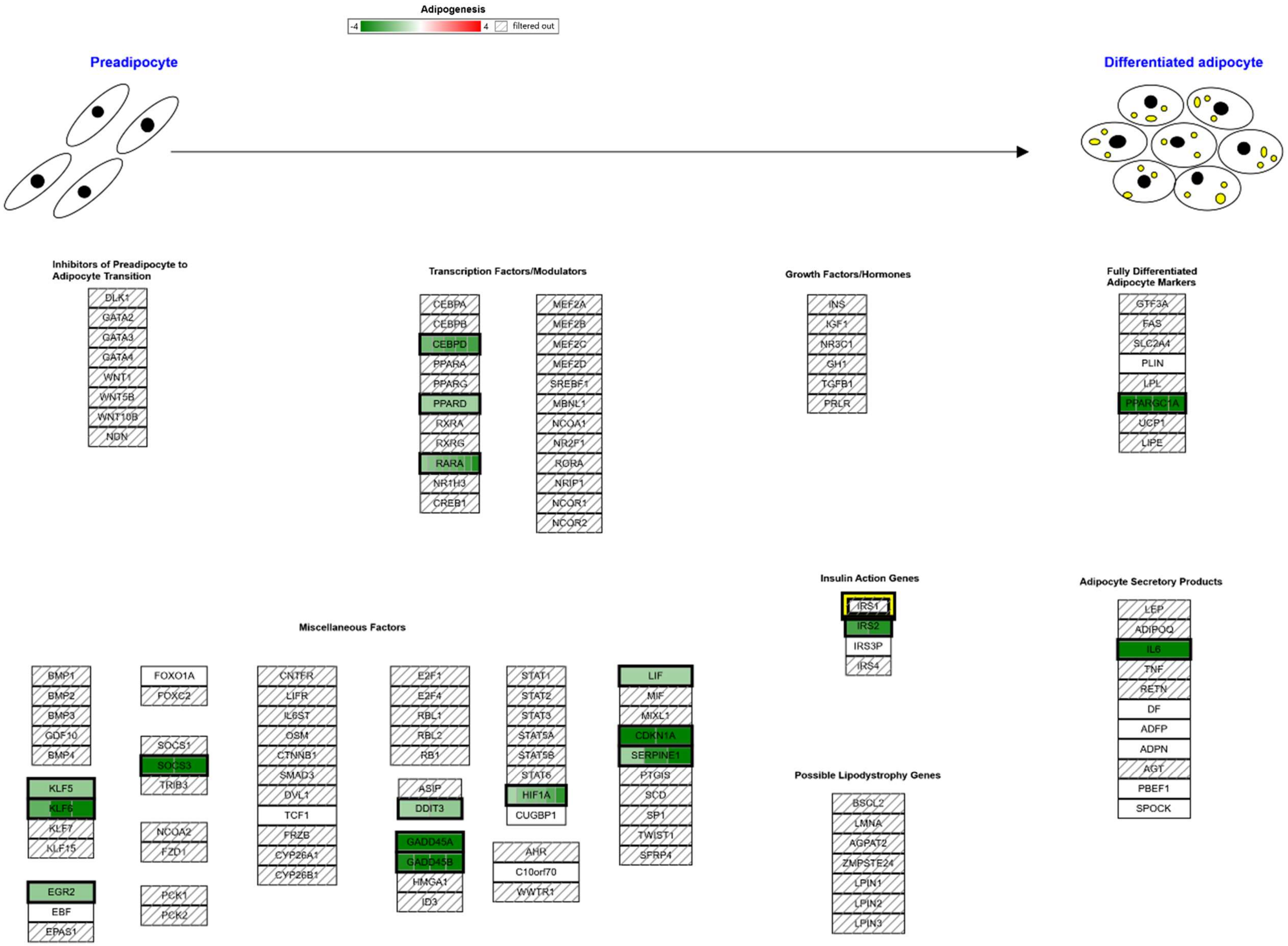

Figure S5: Adipogenesis (Homo sapiens) pathway provided by Wiki-Pathways (https://www.wikipathways.org/index.php/Pathway:WP236). Green marked genes signify a fold change < -4.
